# Deficiencies in the Risk Assessment of Genetically Engineered Bt Cowpea Approved for Cultivation in Nigeria: A Critical Review

**DOI:** 10.3390/plants11030380

**Published:** 2022-01-29

**Authors:** Christoph Then, Juliana Miyazaki, Andreas Bauer-Panskus

**Affiliations:** Testbiotech e.V., Institute for Independent Impact Assessment of Biotechnology, 80807 Munich, Germany; info@testbiotech.org (J.M.); panskus@testbiotech.org (A.B.-P.)

**Keywords:** Bt cowpea, transgenic plants, environmental risk assessment, biodiversity, gene flow, food safety, GMOs, LMOs, Cartagena Protocol on Biosafety

## Abstract

We analyze the application filed for the marketing and cultivation of genetically engineered Bt cowpea (event AAT 709A) approved in Nigeria in 2019. Cowpea (*Vigna ungiguiculata*) is extensively grown throughout sub-Saharan Africa and consumed by around two hundred million people. The transgenic plants produce an insecticidal, recombinant Bt toxin meant to protect the plants against the larvae of *Maruca vitrata*, which feed on the plants and are also known as pod borer. Our analysis of the application reveals issues of concern regarding the safety of the Bt toxins produced in the plants. These concerns include stability of gene expression, impact on soil organisms, effects on non-target species and food safety. In addition, we show deficiencies in the risk assessment of potential gene flow and uncontrolled spread of the transgenes and cultivated varieties as well as the maintenance of seed collections. As far as information is publicly available, we analyze the application by referring to established standards of GMO risk assessment. We take the provisions of the Cartagena Protocol on Biosafety (CPB) into account, of which both Nigeria and the EU are parties. We also refer to the EU standards for GMO risk assessment, which are complementary to the provisions of the CPB.

## 1. Introduction

Cowpea (*Vigna ungiguiculata*) is a legume that is extensively grown throughout sub-Saharan Africa. Millions of African farmers grow cowpea and some two hundred million Africans consume it. Cowpea is one of the most ancient crops known to man. Its origin and domestication occurred in Africa near Ethiopia and was subsequently developed mainly in the farms of the African Savannah. Nowadays, it is a legume widely adapted and grown throughout the world [1]. At the same time, Nigeria is one of the centers of origin maintaining the largest cowpea germplasm collection, with more than 15,000 landraces and over 2000 wild relatives [2].

Genetically engineered cowpea was approved for cultivation in Nigeria in 2019 [3]. The transgenic plants produce an insecticidal recombinant (r-)Bt toxin (originally derived from soil bacteria, *Bacillus thuringiensis*). This toxin is meant to protect the plants against the larvae of *Maruca vitrata* (Lepidoptera: Crambidae), which is a pod borer feeding on cowpea.

Our attention was drawn to Bt cowpea as it is the first transgenic legume to be grown on a large scale in Africa. Cowpea is also traditionally grown on other continents such as Asia, South America and Europe. It is, therefore, worthwhile to see which hazards were identified in these locations and how they were dealt with in the risk assessment. However, we encountered some obstacles in our research, e.g., risk assessment data categorized as confidential business information. 

We analyzed the application for the marketing and cultivation of the insect-resistant cowpea event AAT 709A [4]. We assessed the publicly available information and data, taking into account the provisions of the international Cartagena Protocol on Biosafety (CPB), of which both Nigeria and the EU are parties. According to the CPB, “the objective of risk assessment, under this Protocol, is to identify and evaluate the potential adverse effects of living modified organisms (LMO) on the conservation and sustainable use of biological diversity in the likely potential receiving environment, taking also into account risks to human health” (Annex III, Objective of [5]). We also refer to the EU standards of GMO risk assessment [6], which are complementary to the provisions of the CPB and in line with the Nigeria biosafety guidelines [7].

Our analysis showed two major issues of concern in regard to the risk assessment performed and concluded in Nigeria: (i) the safety of the Bt proteins produced in the plants and (ii) the potential environmental spread of the genetically engineered plants, including gene flow. Given the importance of cowpea for the diets of millions of Nigerians, our assessment also includes risks at the stage of consumption. We identify and describe potential hazards and risks which should have been taken into account in the GE cowpea risk assessment. 

## 2. Results

The analysis of the available information led to the identification of deficiencies in the safety assessment of the Bt toxins produced in the plants. These concerns include: the expression of the additionally inserted genes, the impact on non-target organisms, and the impact on bio-geochemical processes and food safety.

### 2.1. Gene Expression of the Inserted Genes 

Article 15 and Annex III of the CPB request data on the recipient and the donor organism, the vector and the inserts [5]. Regarding the methodology, the CPB requests “an identification of any novel genotypic and phenotypic characteristics associated with the living modified organism that may have adverse effects on biological diversity in the likely potential receiving environment, taking also into account risks to human health” (page 28 of [5]). According to EU standards, which reflect the provisions of the CPB, data on the expression of the inserted genes are part of the molecular characterization and a starting point for risk assessment [6,8]. 

#### 2.1.1. Relevant Issues for Risk Assessment 

*Agrobacterium tumefaciens* was used for the transformation of the recipient plant. It is, therefore, not possible to predict the insertion site, the number of inserted copies, or the final structure of the inserted DNA [9]. Consequently, each event resulting from the transformation has to be assessed case-by-case, taking into account the gene construct, the number of inserted copies, the insertion site, the expression of the transgene, and the stability of its function as well as its inheritance in offspring generations. The genetic engineering techniques can cause additional specific unintended effects [9,10,11,12,13,14,15], including epigenetic effects [16]. As a result, these unintended effects also have to be assessed on a case-by-case basis and cannot be deduced from other events. Moreover, experience gained from conventional breeding cannot simply be extrapolated to the risk assessment of GE organisms. According to the scientific literature [17,18,19,20,21,22,23,24,25], the expression of r-Bt genes can, for example, be impacted by the genetic or epigenetic background of the varieties into which the transgenes are inserted, by interactions with environmental factors and stressors or by the different stages of vegetation (i.e., growing, flowering, ripening). As far as hazards are concerned, unintended effects caused by the insertion of the transgenes may trigger unexpectedly high or low levels of r-Bt toxins in various plant tissues with significant consequences for the environment and food production. Furthermore, the number of gene constructs, the insertion site, and their interaction with the plant genome can cause unintended changes in plant composition and phenotypical characteristics, e.g., seed dormancy, number of pollen and seeds, pollen viability and response to biotic or abiotic stressors. 

#### 2.1.2. Risk Assessment Performed Regarding Gene Expression 

The data submitted by the applicant show that the transgenic cowpea is engineered to express two gene products: in addition to Bt toxins (r-Cry1Ab, the original gene was derived from *Bacillus thuringiensis*), the plants express the enzyme Neomycin phosphotransferase (NptII), which confers resistance to several antibiotics. The data also provide some information on gene expression in the plant. Table 6 (page 34) of the application [4] gives an overview of expression data for r-Bt proteins in the genetic background of two cowpea varieties. Expression data from only one variety were provided for the nptII gene. Data are provided for both of the two varieties (including leaf, flower, pods, green cotyledon and dry seed) that could be used to compare the expression of the r-Bt gene. Raw data were not available, only the mean of replicate samples with the lowest and highest individual values in parentheses (Table 6, page 34, of [4]). At least two groups of the data (flower and pods) point to differences between the two varieties that are likely to be of statistical relevance since their range does not overlap. A third group of data (green cotyledon) is also likely to show significant differences, with only a small overlap range in data from the two varieties. These findings indicate that the expression of the r-Bt gene in the transgenic cowpea is substantially impacted by the genetic background and might, in addition, also be influenced by environmental factors. However, no further data were available for r-Bt gene expression if inserted or crossed into the genome of other varieties, landraces or wild relatives. 

The concentration of r-Cry1Ab toxin measured in the flowers (22,8 µg/g FW) is high compared to the plant tissue of other transgenic plants developed so far (see Table 7, page 35 of [4]). As further data from the applicant show [4], the concentration of the r-Bt toxins varies substantially during the stages of plant growth and maturation. The applicant gives some explanation stating: “Cry1Ab accumulation largely parallels the accumulation of total protein during leaf development and maturation, a result that is consistent with the fact that Cry1Ab expression is driven by the promoter of a gene encoding a major protein constituent of photosynthetically active tissue (small subunit of Rubisco) promoter used to drive expression of Cry1Ab” [4] (p. 80). In addition, the applicant correctly explains that the promoter which controls the expression of the r-Bt proteins in the transgenic plants (the Arabidopsis Rubisco Small Subunit promoter) is known to be influenced in several plant species by signaling pathways involving abscisic acid and jasmonates [26]. The phytohormone, jasmonate, and its derivates have important roles as signaling molecules in plant defense, particularly against insect herbivores [27,28]. If these signaling pathways interact with r-Bt gene expression, then environmental factors, such as biotic and abiotic stressors, may substantially influence Bt gene concentration in the transgenic plants. The applicant is aware of this problem and states that “there is little evidence available to indicate that under field conditions the Rubisco Small Subunit promoter is significantly affected by environmental factors” [4] (p. 80). However, the absence of conclusive evidence is not sufficient to disregard this concern. Based on the data presented, it has to be assumed that the r-Bt content in the plants can be significantly impacted by the genetic background as well as by environmental conditions during cultivation. 

In our opinion, further data should have been requested to conduct an in-depth risk assessment. However, the National Biosafety Committee (NBC) did not see the need to request further data [29]. 

### 2.2. Impact on Non-Target Organisms 

As mentioned, Annex III of the CPB requests the identification of characteristics associated with LMO that may have adverse effects on biological diversity in the likely potential receiving environment [5]). According to EU standards, potential adverse effects of the GE plants on biodiversity and its functioning at several levels have to be assessed. In particular, interactions with non-target organisms (NTOs) have to be taken into account in environmental risk assessment. These NTOs include pollinators, beneficial insects as well as protected species, and may also include wildlife species feeding off the plants. It is not only the managed terrestrial agro-ecosystem that should be taken into consideration, but also their margins and the wider environment [6]. 

#### 2.2.1. Relevant Issues for Risk Assessment 

The selectivity and efficacy of the r-Bt toxins introduced and expressed in plants can be extensively impacted and modified in comparison to Bt toxins from natural sources [30,31,32,33]. Relevant factors here include the structure of the toxin and the combination with other stressors or plant components. Furthermore, the expression of Bt toxins in the plant tissue results in new paths of exposure and accumulation, including changes in quality and quantity when compared to the natural variants of Bt toxins (for overview see [34]). In addition, with cowpea, plant compounds, such as the protease inhibitors (PI) produced in the plants, can cause Bt toxins to degrade more slowly than in isolation. This can result in higher toxicity of the r-Bt toxins if taken up together with the plant tissue when compared to toxins in isolation [35,36,37,38,39]. Indeed, there is evidence that the toxicity of Bt toxins is increased if combined with PI [40,41,42,43,44]. It is known that co-factors that enhance the toxicity of Bt proteins can also impact their selectivity (for overview, see [32]): if synergistic or additive effects occur that increase the efficacy of Bt toxins, its selectivity may be decreased and a wider range of non-target organisms may become susceptible. Some publications do indeed indicate effects of PI combined with Bt toxins on non-target insects, which will need further research [45,46,47,48]. Apart from this, there are several modes of action under discussion for Bt proteins. As [12,32,49] show, there are complex modes of action that have to be taken into account. 

These findings need to be carefully considered during risk assessment. They show that a very detailed risk assessment is needed to address potential adverse effects in non-target organisms. As a result, due to the complexity outlined, risk assessment of Bt cowpea cannot be concluded by testing the protein in isolation, or from data stemming from other events, or other plant species, but requires a case-specific approach. The OECD provides in Table 8 a list of non-pest arthropods associated with cowpea, including many pollinating and beneficial species that are natural enemies of cowpea pests and general predators [50] (p. 34). In regard to the food web, it has to be taken into account that Bt toxins might accumulate in higher concentrations within the tiers of the food web, especially if taken up by predatory insects, e.g., beneficial predator wasps feeding on the larvae of *Maruca vitrata* [51,52,53,54]. An extensive list of parasitoids and entomoviruses that attack the pod borer, *Maruca vitrata*, in West Africa can be found in Table 7 in OECD [50] (p. 34). As stated in [50], in addition to parasitoids, generalist predators also feed on cowpea insect pests. These include mites, beetles, ants, bugs and spiders. Therefore, a list of organisms in Nigeria that could potentially be exposed, directly or indirectly, to the plant material should have been provided before any field trials were authorized or cultivation allowed. These organisms should be subjected to specific tests in the laboratory, or a greenhouse, to provide the relevant data on the toxicity and impact of the plant material. The investigations should include synergistic effects of Bt toxins and PI as described and evidenced [35,36,37,38,39,40,41,42,43,44]. As far as hazards are concerned, the effects on non-target organisms need to be fully investigated, as plant material from the Bt cowpea may have unexpected and severely adverse effects on ecosystem services, the food web and biodiversity of exposed insects or wild species, e.g., birds or other non-target organisms.

#### 2.2.2. Risk Assessment Performed on Non-Target Organisms

As explained above, several modes of action of Bt proteins have been under discussion [30,31,32,49]. As a result, a broader range of potentially affected organisms has to be taken into account than was originally assumed [34,55]. However, the application [4] (see also the diagram on page 53) only refers to one single mode of action. Further, the applicant makes no acknowledgement of the fact that not all the steps for activation as observed with natural toxins are necessary for the ones produced in the r-Bt cowpea. Consequently, the mode of action of Bt toxins presented by the applicant is not only outdated, but also misleading: “The spectrum of insecticidal activity of Cry1Ab is extremely narrow, with activity only against Lepidoptera [43]. The insecticidal specificity of Cry1Ab is the result of the numerous essential steps involved in producing an active protein toxin and its subsequent interaction with the epithelial cells in the insect midgut. To exert its insecticidal activity, Cry1Ab must: (i) be ingested by the insect and solubilized in the insect gut, (ii) be activated by specific proteolytic cleavage by insect midgut enzymes, (iii) bind to specific receptors on the surface of the insect midgut, and (iv) form ion channels in the gut membrane. The completion of all four of these processes results in damage to the insect midgut, leading to gut paralysis and death of the insect [56]. The series of events leading to toxicity against Lepidopteran insects is highly specific to Lepidoptera, and does not occur in mammals, other vertebrates or other orders” [4] (p. 52). 

Indeed, the data submitted by the applicant show that the r-Bt toxins expressed in the plants are not identical to Bt toxins found in natural soil bacteria. Rather, it was modified by Monsanto to improve its expression in the plant cell [57]. As described in the application, “the nucleotide sequence of the cry1Ab gene was codon-optimized for plant expression and encodes a 615-amino acid protein (68.9 kDa) corresponding to the trypsin-resistant insecticidally active core protein following cleavage of the 1155-amino acid native Cry1Ab protoxin” [4] (p. 31). In other words, the naturally occurring bacterial Bt pro-toxin was truncated so that the Bt plants produce the toxin in its activated form. Therefore, the first step in the activation of the toxin, which takes place naturally in the intestine of the insects, is not needed for the Bt toxins produced in the plants. It is important to note that this activation is crucial for the specificity of Bt proteins [58]. Therefore, changing the toxin’s structure might have changed its selectivity and efficacy as well, rendering it toxic for a broader range of non-target organisms [30,31,32,33]. Besides the truncation, there are further changes in the structure of the r-Bt proteins produced in the plants [57]. It is not clear from the data presented by the applicant if this specific r-Bt protein is produced in any other transgenic plants. 

As the citation above shows, the applicant is also aware of the findings of MacIntosh et al. [43], who report higher toxicity of Bt toxins if these are combined with PI produced in cowpea. However, the reference to the publication was not integrated into the reference list in the application. Whatever the case may be, despite being aware of relevant publications showing specific risks, the applicant did not deliver any data to demonstrate the safety of the Bt cowpea in regard to non-target organisms. Instead, the applicant refers to publications reporting experiments with non-target organisms from other continents, not involving materials from the Bt cowpea (such as those mentioned in [59] or [60]). It is plausible that the synergistic effects described by [40,41,42,43,44] may also impact non-target organisms and the food webs. As mentioned, there are some publications indicating effects of PI combined with Bt toxins on non-target insects, which will need more research [45,46,47,48]. However, no data are presented on the wide range of organisms that interact with the Bt cowpea, including insects, mites, nematodes and parasitic plants as well as birds, rodents and other mammals, which feed on or interact with the plants. None of the relevant species (apart from *Maruca vitrata*) that are abundant in Nigeria was tested in a targeted study. There seems to be a general lack of empirical risk assessment studies. A more recent Bt cowpea study [61], which was compiled by experts who had already contributed to [59], does not mention any empirical testing of non-target organisms that are specific to Nigeria.

In summary, the applicant did not acknowledge empirical findings showing toxicity of r-Bt toxins beyond the expected range of organisms. In addition, the existing scientific evidence on the factual complexity of the mode of action of Bt toxins was not taken into account. Furthermore, the applicant did not take into account the existing evidence showing an increase in the toxicity of Bt proteins if these are combined with PI. It is likely that a broader range of non-target organisms will be affected by the cultivation of the Bt cowpea than assumed by the applicant. This can severely impact ecosystems and biodiversity. However, the National Biosafety Committee (NBC) did not request additional data, which would have been necessary to derive substantiated conclusions in regard to non-target organisms, nor did it call the statements made by the applicant into question [29]. 

### 2.3. Impact on Bio-Geochemical Processes

As mentioned, Annex III of the CPB requests the identification of characteristics associated with LMO that may have adverse effects on biological diversity in the likely potential receiving environment [5]). According to EU standards, plant-associated and soil microbial communities (e.g., rhizosphere) perform vital biotransformations for sustainable soil fertility, and therefore any negative impact(s) on these organisms should be carefully evaluated on a case-by-case basis, with particular reference to the characteristics of the introduced trait and the consequences of genetic alteration in the GE plant [6].

#### 2.3.1. Relevant Issues for Risk Assessment 

Publications on field trials with other Bt crops show that changes in the microbial community are to be expected [62,63,64,65,66,67,68]. Experiments showing a delay in the degradation of Bt toxins in the soil if combined with cowpea trypsin inhibitor [63] are especially relevant in this context. The presence of this cowpea PI significantly delayed the degradation of r-Bt proteins produced by the transgenic cotton. Under these conditions, the repeated cultivation of transgenic cotton had considerable negative effects on the microbial properties and enzymatic activities in rhizosphere soil compared to those in the rhizosphere soil of non-transgenic cotton [63]. As far as hazards are concerned, there needs to be an assessment of repeated cultivation of transgenic cowpea, as this could have negative effects on the microbial properties and enzymatic activities in rhizosphere soil, thus affecting soil fertility and plant health. 

#### 2.3.2. Risk Assessment Performed on Bio-Geochemical Processes 

Cowpea has unique symbiotic relations with specific communities of soil microorganisms (i.e., rhizobia, mycorrhizae) [50]. It is also known that cowpea is a symbiont to specific endophytes (see, for example, [69]). Furthermore, r-Bt toxins are also produced in the roots and, additionally, plant material, such as that left after the harvest, will also contribute to additional r-Bt toxins in the soil. However, this was not considered and no empirical tests were conducted by the applicant to assess changes in the associated endophytes, or other communities within the plants’ microbiome, or in the soil rhizosphere. Neither was any data presented on the wide range of microorganisms that interact with the Bt cowpea, including symbionts (e.g., mycorrhizae, rhizobia, endophytes) or pathogens (bacterial, viral and fungal microorganisms). Instead, the occurrence of soil bacteria naturally producing Bt toxins is used by the applicant to claim that a specific risk assessment would not be needed. While it is true that some soil bacteria do produce Bt toxins naturally (see, for example, [70]), these findings do not imply that the influence of r-Bt toxins produced by the transgenic plants on microbial communities does not need to be investigated during risk assessment. There are several reasons: investigations of Bt cotton and Bt corn show that transgenic plants produce a much greater amount of Bt toxins compared to soil microorganisms [71]. Compared to Bt toxins produced by soil bacteria, r-Bt toxins produced in the cowpea differs extensively in structure and pattern of exposure to the environment [34]. Furthermore, Bt toxins naturally occurring in soil bacteria are not produced in combination with PI as is the case with the Bt cowpea. Experiments showing a delay in the degradation of Bt toxins in the soil if combined with cowpea trypsin inhibitor [63] are especially relevant in this context (see above). Therefore, empirical testing of the relevant microbial species, including the plant microbiome and long-term impact on the soil rhizosphere, is necessary before concluding on the environmental safety of Bt cowpea. The experiments should also take into account synergistic effects such as those made evident by several publications [35,36,37,38,39,40,41,42,43,44]. 

In summary, we are of the opinion that the environmental safety of Bt cowpea was not demonstrated since no data were provided by the applicant on its impact on the relevant microbial communities. It is not unlikely that r-Bt toxins expressed in the plants can adversely impact microbiomes, including endophytes, mycorrhizae and other soil organisms. The National Biosafety Committee (NBC) did not see the need to request further data [29]; it was possibly not aware of the specific hazards triggered by the combination of PI and the r-Bt toxins on bio-geochemical processes. 

### 2.4. Food Safety 

Article 15 and Annex III of the CPB request that risks to human health also be taken into account. According to EU GMO regulation, the toxicological impact of any changes in whole food and feed resulting from genetic modification, such as the introduction of new genes, gene silencing or over-expression of an endogenous gene, must be assessed. In addition, the applicant must also provide an analysis of key toxins inherently present in the recipient plant, which may adversely affect human or animal health as well as anti-nutritional compounds, such as digestive enzyme inhibitors and already identified allergens [8].

#### 2.4.1. Relevant Issues for Risk Assessment 

As mentioned above, there are many publications showing that Bt toxins have several modes of action which impact a broad range of non-target organisms, irrespective of the boundaries of taxonomy. This is also the case with Cry1Ab. Furthermore, selectivity and efficacy can be influenced by co-factors and changes in the structure of the r-Bt proteins. In the case of cowpea, plant compounds, such as PI, can cause the r-Bt protein to show higher toxicity than it would in isolation. As shown, there are specific findings evidencing an increase in the toxicity of Bt proteins if combined with PI present in cowpea [35,36,37,38,39,40,41,42,43,44]. Such synergetic effects not only enhance the effectivity (toxicity) of Bt toxins but may also impact their selectivity (for overview, see [32]). Whatever the case may be, these synergistic effects are likely to result in a higher toxicity of Bt toxins if these are taken up together with the plant tissue, when compared to the toxin in isolation (see also [72]). It is evident that neither feeding studies using the isolated protein nor feeding studies using other Bt staple food, such as maize or soybean, are reliable when assessing the potential health effects of Bt cowpea at the stage of consumption. Whole plant feeding studies should instead be conducted with Bt cowpea. In this context, there is also some cause for concern that Bt toxins can trigger non-allergic immune responses, e.g., adjuvant effects [73,74,75,76,77,78,79,80,81,82,83,84], which might contribute to chronic diseases or enhance immune responses. It is largely acknowledged that more data are needed on adjuvant and other potential immune responses caused by Bt proteins (see, for example, [82,84]. The synergistic effects between Bt proteins and PI causing higher toxicity of the Bt toxins are also relevant to risk assessment in regard to the immune system: the combination with PI is likely to be associated with a delay in the degradation of the Bt toxins after consumption. In regard to hazards that need to be assessed, this delay in degradation extends the exposure of the intestinal immune system to Bt toxins, and may trigger or enhance chronic inflammation and allergies.

There are additional and specific reasons why the Bt toxins (r-Cry1Ab) expressed in the cowpea should undergo detailed testing for potential immune responses: according to [85] and [86], some proteins naturally produced in cowpea beans are regarded as potential allergens. The occurrence of some allergies has been identified, although these are still rare for cowpea: research published in 2000 investigated serum from six patients allergic to cowpea 41 kDa and 55 kDa proteins, which were identified as cowpea allergens [86]. These proteins were detected in subspecies of *Vigna unguiculata* grown in Asia (*Vigna sinensis*), which are thought to have a common origin with the African subspecies [87]. Thus, combining these proteins with immune reactive Bt proteins might foster allergenic allergies via adjuvant effects. In light of the findings on synergistic effects with PI (see above), it also has to be considered that in raw or less processed cowpea used for human consumption, the toxicity and potential immune effects of Bt proteins are likely to be significantly higher compared to heated beans, as heat may partially deactivate PI and Bt toxins. This needs to be carefully addressed in risk assessment. 

#### 2.4.2. Risk Assessment as Performed in Regard to Food Safety 

The applicant refers to mouse toxicity studies that aimed to assess short-term high exposure to isolated Bt proteins. However, such studies are of little value as these experimental conditions do not allow identification of synergistic effects, which are likely to occur in the plant material and which might cause higher overall toxicity in the diet. The effects caused by the PI described by [35,36,37,38,39,40,41,42,43,44] that leads to higher toxicity of Bt toxins show that the approach chosen by the applicant is inadequate. 

The studies indicating rapid degradation of the Bt toxins after ingestion referred to by the applicant are contrary to other findings: Chowdhury et al. [88] and Walsh et al. [89] found that r-Cry1A proteins could frequently and successfully still be found in the colon of pigs at the end of digestion when they were fed with Bt maize. Thus, the r-Cry1A proteins can show higher stability, at least in monogastric species, than predicted by digestion experiments using isolated Bt proteins. In this context, it is important to note that plant compounds in cowpea, such as the PI, can cause the Bt toxins to degrade more slowly than, for example, in maize. Therefore, higher exposure to the r-Bt toxins after consumption has to be expected if it is taken up together with the plant tissue in comparison to the toxin in isolation. The delay in degradation also causes higher r-Bt toxin exposure of the immune system via the intestinal tract. Under these conditions, immune responses, such as chronic inflammation and allergies, may be triggered or enhanced (see also [72]). As shown above, specific experimental evidence found that cowpea produced potential allergens [86]. However, no similar specific and experimental investigations were conducted by the applicant [4]. Instead, the applicant [4] refers to the AllergenOnline database [90], without any discussion of the specific findings of Rao et al. [86], also referenced by OECD (2018) [85]. Therefore, it cannot be excluded that combining allergenic proteins with immune reactive Bt proteins might foster allergenic reactions via adjuvant effects. 

In summary, a detailed investigation of potential health effects at the stage of consumption is absolutely essential and should take into account all traditional usages, including diets with raw cowpea ([85], see also below). However, the applicant did not even perform a single feeding study with the whole food and feed derived from the Bt cowpea. It is astonishing that National Biosafety Committee (NBC) did not request more data from the applicant, for example, in regard to findings on potential allergens.

### 2.5. Gene Flow to Other Cultivated Varieties or Wild Relatives 

Annex III of the CPB requests information on “the biological characteristics of the recipient organism or parental organisms, including information on taxonomic status, common name, origin, centres of origin and centres of genetic diversity, if known, and a description of the habitat where the organisms may persist or proliferate” (page 29 of [5]). According to EU standards, the potential persistence or invasiveness of the GE plant itself, or of its compatible relatives, as a result of gene flow within either agricultural or other production systems, or semi-natural and natural habitats, is an issue especially relevant to environmental risk assessment [6]. 

#### 2.5.1. Relevant Issues for Risk Assessment 

The *Vigna unguiculata* species complex is currently divided into eleven subspecies. Ten of the subspecies are perennial and one subspecies is annual [50]. There are no apparent barriers to hybridization, or recombination between members of the different cultivar groups or with the wild cowpeas (var. *spontanea*) in the subspecies unguiculata [50]. As stated in OECD (2015): “The overall message is that crosses appear possible among all members of the *Vigna unguiculata* complex, but they vary from being easy to being difficult" [50] (p. 25). Wild relative subspecies belonging to the group *Vigna unguiculata* var. *spontanea* are known to occur in Nigeria as well as in many other West African countries. *Vigna unguiculata* var. *spontanea* can be found mostly in disturbed areas (such as fields, field margins, roadsides and fallows) but also in natural ecosystems, such as those observed in Cameroon, Uganda and Ethiopia [91]. While often considered a rare event, gene flow was observed between cultivated and wild relatives in each of the populations investigated [92]. As summarized in OECD 2016 [91], cultivated cowpea readily crosses with wild cowpea in the same subspecies (i.e., var. *spontanea*) and can be crossed with members of the other subspecies of *Vigna unguiculata*, albeit with varying degrees of difficulty.

There are several pollinating insects involved in the distances and success rates of gene flow, but in many cases, their specific role still needs to be investigated. As stated in OECD (2015): "Cross-pollination is usually less than 1% but will vary somewhat with the cultivar and, more particularly, with the population of some insects. In several cases, the pollinators are not known, but honeybees (*Apis mellifera*) have been observed around cowpea flowers and thus have been implicated in pollination (…) In coastal Kenya and Burkina Faso, several large carpenter bee species (*Xylocopa* spp.) and leafcutter bee species (*Megachilidae* spp.) were considered potential cross-pollinators of cowpea (...), and it was shown that these same leafcutters and carpenter bees were the likely pollinators of the wild progenitor of cowpea (…) Casual observations made in California, Texas and Nigeria indicate that large bumblebees (*Bombus* spp.) may be responsible for the cross-pollination that occurs in cowpeas in these regions” [50] (p. 22). Under these conditions, gene flow is very likely to occur in the fields when the GE cowpea is grown in close vicinity to regional varieties and without sufficient distance to wild relatives in other bio-geographical zones. For example, as NEPAD’s African Biosafety Network of Expertise [93] explains on their website, 90 % of the pollination in cowpea stems from self-fertilization. Consequently, there is still a significant possibility of gene flow occurring within the remaining 10 %. Therefore, it is likely that the cultivation of the Bt cowpea will lead to the introduction of the recombinant genes into regional varieties, whether intentionally or unintentionally. Gene flow to wild relatives is also very likely to occur in the longer term. This finding is supported by Huesing et al. and reflects the perspective of experts affiliated with the biotech industry. In regard to the gene flow of Bt cowpea, these experts state: “Based on existing information, the panel determined that hybridization is likely to occur” [59] (p. 214). Wild cowpea plants are often not uprooted from the field, and appear to be tolerated in the agroecosystem. The hybrid progenies may even end up being used by farmers for sowing, and may be considered as fodder landraces [91]. This poses substantial risks for the protection of natural biodiversity and the maintenance of landraces and regional varieties which might become contaminated by pertinent gene flow or seed contamination from Bt cowpea. This risk is acknowledged by the International Institute of Tropical Agriculture (IITA) in its conservation strategy for genetic resources of cowpea and its wild relatives, which proposed urgent measures especially for Nigeria: “Collecting missions should take place in the following four high priority regions: (…) Nigeria for wild cowpea (mainly subsp. *unguiculata* var. *spontanea*), as it is underrepresented in ex situ collections. Moreover, there is a risk of genetic contamination from the introduction of Bt cowpea in the country (field trials started in 2009)” [94] (pp. 7–8). 

Therefore, due to gene flow, the transgenes will be introduced into plants with largely heterogeneous genetic and epigenetic backgrounds. There is evidence that the biological characteristics of the offspring generation in many cases cannot be predicted from the original genetically engineered event [95,96,97,98,99,100]; for overview see [101]. If the offspring can persist and propagate in the environment, interactions with the environment or changes in the environmental conditions can, in addition, play a major role in triggering unintended biological effects [24,25,102,103,104,105,106]. As far as hazards are concerned, issues such as, e.g., changes in the plant composition of the hybrid offspring, need to be addressed. This can involve changes in their metabolism and signaling pathways, impacting their interaction with pollinators or associated soil organisms in a way that can impact essential ecosystem services. Other examples of potential adverse effects include the higher vulnerability of the plants’ offspring to stressors, or enhanced gene flow and spread into the environment causing potential invasiveness. Furthermore, effects observed in hybrid offspring may include unexpectedly high or low levels of r-Bt toxins produced in the various tissues of the plants. 

#### 2.5.2. Risk Assessment Performed in Regard to Gene Flow 

As Figure 1 (page 19) of the dossier shows [4], wild relatives (var. *spontanea*) of domesticated cowpea grow in the same regions where cowpea is cultivated. Under these conditions, there is a high likelihood that viable hybrid offspring will occur not only in the fields but also in adjacent areas. The applicant states that “the ecosystem into which AAT 709A could be disseminated is exactly the same as for conventional cowpea, namely managed agricultural environments where cowpea is being cultivated, and areas adjacent to managed agricultural environments” [4] (p. 50). Consequently, the exposure of traditional varieties, landraces and wild relatives (var. *spontanea*) to gene flow from Bt cowpea will not be limited in spatial or temporal dimensions. However, the applicant does not consider this to be a problem since reference is made to experiences from field trials: “Apart from the presence of the insect resistance trait, the similarity of event AAT 709A to conventional cowpea based on extensive analysis including molecular characterization, protein expression, agronomic and phenotypic evaluation suggests that AAT 709A would not be expected to have any unintended fitness enhancing traits as a result of the modification. While it is possible that the insect resistance trait could confer a selective advantage to cowpea or wild cowpea that has acquired the trait under specific conditions of: (i) high insect pressure and (ii) presence of natural competitor plants that are significantly controlled by Maruca. As with conventional cowpea it is highly unlikely that event 709A would adversely affect the environment through persistence or invasiveness” [4] (p. 38). 

This statement is based on several assumptions, but not on sufficient evidence: the data derived from the field trials do not include any investigations of spontaneous crossings of the Bt cowpea with more heterogeneous backgrounds, as would be the case if hybridization with regional varieties or wild relatives were to occur. No experimental crossings were performed with *Vigna unguiculata* var. *spontanea*. Instead, the applicant presented data on the genetic stability of the Bt cowpea “prepared from progeny plants spanning ten selfing generations in direct line of descent from the original transformant” [4] (p. 21). Further, the applicant reports on crossings of individual plants from two segregating generations in three genetic backgrounds only. By taking into account the findings on next generation effects, which cannot be predicted from the characteristics of the original event (for overview see [101]), the assumptions made by the applicant have to be called into question. The claims by the applicant that there would be no threat of gene flow or potential spread of the Bt cowpea to biological diversity, traditional crops, farmers’ varieties and sustainable agriculture, are not sufficiently supported by the data and published evidence.

In summary, the consequences of gene flow for biodiversity, seed collections, seed saving and traditional farming were not sufficiently taken into account by the applicant. There is a substantial risk that cultivation of the Bt cowpea may endanger biodiversity in one of the centers of origin of cowpeas. It is very likely that National Biosafety Committee (NBC) was aware of the hazards to biodiversity, gene banks and landraces. Therefore, it should have been a priority to request much more detailed risk assessment [29].

## 3. Discussion

We analyzed the application for marketing and cultivation of insect-resistant cowpea—Event AAT 709A—and the respective recommendation of the National Biosafety Committee (NBC) of Nigeria [3]. In this context, it is a generally accepted principle that genetically engineered organisms (or LMOs as they are referred to by the CPB) can only be released into the environment if adequate risk assessment is performed beforehand. To be considered adequate, risk assessment must be based on sound science, and take intended and unintended effects into account. It should, moreover, be sufficiently robust and conclusive. This is necessary to address the precautionary principle underlying the Convention on Biological Diversity and the CPB. Furthermore, the objective of risk assessment under the Protocol is to identify and evaluate the possible adverse effects of LMOs on the conservation and sustainable use of biological diversity, also taking into account risks to human health.

According to the CPB, “the objective of risk assessment, under this Protocol, is to identify and evaluate the potential adverse effects of living modified organisms on the conservation and sustainable use of biological diversity in the likely potential receiving environment, taking also into account risks to human health” (page 27 of [5]). The requirements for carrying out the risk assessment are listed in Article 15 and Annex III of the Protocol. In this context, uncertainties also need to be addressed: “Where there is uncertainty regarding the level of risk, it may be addressed by requesting further information on the specific issues of concern or by implementing appropriate risk management strategies and/or monitoring the living modified organism in the receiving environment.” (page 29 of [5]) 

The provisions of the Protocol also address the tasks of the risk manager and require “an estimation of the overall risk posed by the living modified organism based on the evaluation of the likelihood and consequences of the identified adverse effects being realized.” (page 29 of [5]). 

Finally, Article 16 of the CPB requests risk management strategies that are “appropriate mechanisms, measures and strategies to regulate, manage and control risks identified in the risk assessment provisions of this Protocol associated with the use, handling and transboundary movement of living modified organisms” (page 12 of [5]. 

In order to address these provisions more specifically, we also referred to standards used in EU risk assessment. The standards applied by the EU in the risk assessment of GMOs, can be regarded as an implementation of the CPB, of which the EU is a party. Therefore, these standards can be seen as complementary to the CPB and are also in line with the Nigeria biosafety guidelines [7]. The issues presented and discussed here are also addressed in GMO regulation in other regions, and can also be relevant for other countries in which these GE cowpeas are approved or where applications have been filed for release and marketing, e.g., Ghana, which is also a party to the CPB. Our analysis is based solely on the presented findings and reasoned considerations. 

### 3.1. Molecular Data and Gene Expression 

Cowpea is cultivated in several bio-geographical zones in Nigeria. Some field trials were conducted between 2009-2015 (see [4], Table 8), but no specific ‘stress tests’ were performed under defined conditions followed by detailed analysis of the interactions between the genome and the environment (see, for example [23]). Therefore, the existing evidence of transgenic cowpea responses to specific environmental conditions is very limited. Risk assessment cannot, therefore, be regarded as sufficiently reliable and conclusive without showing consistency in gene expression under a broad range of environmental conditions. On the contrary, based on the data presented, it has to be assumed that sufficient stability in gene expression is lacking. Furthermore, it is likely that the genetic background and environmental conditions have a significant impact on the r-Bt content. In general, unintended changes affecting gene expression, plant composition, metabolism, signaling pathways and response to environmental conditions can affect agro-ecosystems, food production and food safety as well as organisms responsible for essential ecosystem services, e.g., pollinators, associated soil organisms and biodiversity. More specifically, instability in the expression of the additionally inserted genes may, for example, trigger unexpectedly high or low levels of the r-Bt toxins in the various plant tissues, with significant consequences for the environment and food production. An insufficient level for a “high-dose approach” inducing a high degree of lethality in heterozygous individuals carrying single alleles for recessive mutations for resistance against r-Bt effects can cause the plants to be insufficiently protected against infestations of *Maruca vitrata* larvae. Under these conditions, the pest insects can evolve more rapidly to become resistant to the expressed r-Bt toxin [107]. A high concentration of r-Bt toxins, on the other hand, is relevant for assessing risks to non-target organisms and food safety. Moreover, if consistency in gene expression cannot be shown and gene expression is highly impacted by environmental conditions or interactions within the genome, this can cause a wide range of unintended and potentially unpredictable effects on plant metabolism and response to environmental conditions. Under such circumstances, risk assessment can hardly result in a favorable outcome. In regard to the assessment of plant composition and agronomic characteristics, no specific conclusions can be presented here. The applicant did not report any relevant differences between the GE cowpea data compared to the conventional plants, and we did not have access to the data. In general, the design of field trials, the environmental conditions, the agronomic practices and the choice of comparators may all influence not only the gene expression of the additionally inserted genes, but also the outcome of the comparative assessment of plant composition and agronomic characteristics. Therefore, it is important that the design of the field trials sufficiently represents the conditions under which the plants may be cultivated. 

### 3.2. Impact on Non-Target Organisms 

If the rich biodiversity in Nigeria and the current threats to its conservation [108] are taken into account, these questions are highly relevant to GE cowpea environmental risk assessment. Nigeria has several bio-geographical zones which need to be considered; there are substantial differences between these zones regarding fauna and flora. “Each of these ecosystems has its own unique characteristics of wild fauna, higher and lower floral species and a huge collection of marine and freshwater aquatic species. In species diversity and endemism, Nigeria is highly endowed” [108] (p. 20). There is already a substantial threat to biodiversity: “However, overall, biodiversity in Nigeria is highly threatened due to land-use changes from agriculture and overgrazing, overexploitation of natural resources through extractive actors, invasive species and environmental pollution. According to the IUCN Red list 2013, Nigeria has a total of 309 threatened species in the following taxonomic categories: Mammals (26), Birds (19), Reptiles (8), Amphibians (13), Fishes (60), Molluscs (1), other Invertebrates (14) and Plants (168)” [108] (p. 22). A total of 20,000 insect species, including more than 1000 butterfly species, are reportedly living in the ‘Cross-River-National Park’ [109,110]. More specifically, Nigeria is the center of origin for many endemic species and home to more than a thousand butterfly species belonging to the same group of insects (Lepidoptera) as the target species. Further, it is known that a large number of species interact with cowpea plants [50]. The risks for biodiversity are especially relevant since there is considerable overlap between the centers of biodiversity in Nigeria, especially in its northern and central regions where cowpea is cultivated [1]. In addition, small-scale farmers, in particular, might cultivate Bt cowpea in the highly diverse regions where gene flow is likely to expose ecosystems beyond the fields to r-Bt toxins. 

Whatever the case may be, experience gained from the cultivation of plants into which other constructs have been inserted, or which inherit other events, or belong to other species, e.g., Bt maize and Bt cotton, cannot be used to demonstrate the safety of the Bt cowpea. Nevertheless, the applicant claims that the cultivation of these other Bt crops supports their assumptions on the safety of the Bt cowpea. It is evident that such general claims are not sufficiently substantiated. Clearly, each event must undergo a case-by-case environmental risk assessment, taking into account the molecular data, the biological characteristics of the plants and the receiving environments. It is further evident that data on selectivity and the toxicity of the natural variants of Cry1Ab toxin is not sufficient to demonstrate the safety of the specific variant of the toxin expressed in the GE cowpea. As explained, small changes in the structure of the protein can have a significant impact on its toxicity. Therefore, the statements made by the applicant generally assuming a “narrow and specific toxicity” [4] (p. 20) only affecting “interactions with susceptible Lepidopteran pests that feed upon the plant” [4] (p. 69) are not sufficiently based on science, especially without presenting specific experimental data regarding the r-Bt toxins produced in the plants. It is concerning that no such specific environmental data were presented. Despite the applicant being aware that “cowpeas interact with a wide variety of other organisms including symbionts (mycorrhizae, rhizobia), insects, mites, bacterial, viral, and fungal pathogens, nematodes and parasitic plants” [4] (p. 20), no experimental data are presented to show that these complex interactions and interrelations are not impacted, changed, disturbed or disrupted by the r-Bt protein produced in the cowpeas. 

The need for more detailed risk assessment is strongly supported by the findings of MacIntosh et al. [43], Zhao et al. [44], Fan et al. [41], Gujar et al. [42], Cui et al. [40], who all show the specific risks arising from the combination of Bt toxins with PI produced in cowpea, thus causing higher toxicity of the r-Bt proteins through synergistic effects. However, no empirical data were made available regarding the susceptibility of the species listed, for example, by the OECD [50]. As a result, and because the applicant did not provide any data on relevant non-target organisms that are abundant in the regions where the Bt cowpea will be grown, no conclusion can be made regarding potential impacts on non-target organisms. In addition, it is likely that more species are vulnerable to the r-Bt toxins expressed in the plant than assumed by the applicant. 

### 3.3. Impact on Bio-Geochemical Processes 

As explained above, cowpea has unique symbiotic relations with specific communities of soil microorganisms (rhizobia, mycorrhizae) which enhance the flow of reduced nitrogen and phosphate into the cropping system [50]. Bt cowpea produces r-Bt toxin in the roots and, therefore, the impact on soil organisms in the rhizosphere should have been investigated. It is also known that specific endophytes are symbionts to cowpea (see, for example, 69]). Endophytes and plants often engage in mutualism, with endophytes primarily aiding in the health and survival of the host plant, including issues such as pathogens and disease, water stress, heat stress, nutrient availability, poor soil quality, salinity and herbivory [111]. The diversity of the endophytic community varies with plant species, host genotype, type of tissue analyzed, host age, climatic factors and geographic distribution (see, for example, Farias et al. [112]). Therefore, if the genotype of the cowpea is changed and r-Bt toxins are expressed in all tissues of the plant, the composition of endophytes might also change. Consequently, plant response to the environment might also be altered. Given the crucial role of endophytes for plant health, this may have a detrimental effect on the health of the plants under specific environmental conditions. 

Furthermore, the repeated cultivation of transgenic cowpea may have considerable negative effects on the microbial properties and enzymatic activities in rhizosphere soil compared to those in the rhizosphere soil of non-transgenic cowpea. The reason is the presence of the cowpea PI, which delays the degradation of the r-Bt toxins produced by the transgenic cowpea [63]. However, these effects were not taken into consideration by the applicant. Therefore, it cannot be excluded that repeated cultivation of Bt cowpea will have considerable negative effects on the microbial properties and enzymatic activities in rhizosphere soil. 

### 3.4. Food Safety

The consumption of cowpeas as a main staple food in many regions might result in relatively high exposure of humans to r-Bt toxins when compared to other Bt plants (see Table 7, page 35 of [4]). Therefore, risk assessment should take into account the concentration of the r-Bt toxins and degradation throughout the relevant stages of processing of all parts of the plants (pods, beans and leaves) intended for human consumption. As far as human consumption is concerned, cowpea is mainly grown for grain (dry and fresh) and sometimes also for fresh pods and leaves [85,113]. In general, the green and fresh edible parts of the plants (pods, leaves and beans) will undergo less processing compared to dried beans. Also in this context, it has to be taken into account that there are several bio-geographical zones in Nigeria [109] which are substantially different not only in respect to fauna and flora but also in agricultural and cooking practices and their huge ethnic diversity. For example, in Nigeria, the cooking time of cowpea is traditionally reduced by cooking it with a naturally occurring alkaline rock-salt known as ‘kanwa’ [85]. Furthermore, soaking the cowpea before cooking is also widely used to reduce heating time. These traditional practices may possibly impact the degradation or non-degradation of r-Bt proteins in the diet. It follows that any application for field trials or agricultural cultivation should be accompanied by data on food and feed safety, encompassing long-term (chronic) feeding studies with whole plant food and feed. Fresh material and food that is processed to a lesser extent than dried beans should also have been taken into account. In this context, cowpea is reported to show allergenic potential [86] and Bt proteins are suspected of enhancing or provoking immune responses (see above). Therefore, targeted studies should have been performed to exclude risks for the immune system. Instead, the applicant presents very general statements such as “cowpea is not considered toxic, nor pathogenic to humans, and is not considered allergenic” [4] (p. 36), not mentioning that some proteins with potential allergenicity were identified by experimental research in *Vigna unguiculata* [86]. Furthermore, the applicant does not mention the immunogenic properties of Bt proteins. Consequently, more detailed empirical investigations into the long-term impact of Bt cowpea at the stage of consumption would be needed before risk assessment can be concluded. However, the applicant did not even perform a single feeding study with the whole food and feed derived from the Bt cowpea as requested by EU standards [6]. 

### 3.5. Gene Flow to Other Cultivated Varieties or Wild Relatives 

West Africa is among the regions in the world with the earliest evidence of cowpea cultivation, dating back more than 1500 years [114]. Therefore, cowpea is considered to be a significant part of pan-African heritage that is closely related to the history of countries, such as Ghana, South Africa and Nigeria. While the largest production area is in Africa, also Brazil, West India, Myanmar, Sri Lanka, Australia, the United States, Bosnia and Herzegovina all have significant production [1]. Cowpea is morphologically variable and adapted to different environments, resulting in a wide range of local varieties [50]. The nutritional composition of cowpea is impacted by genetic characteristics, agro-climatic conditions, biotic stresses and postharvest management [85]. Interestingly, despite the considerable morphological diversity, limited genetic diversity occurs among cultivated cowpea varieties owing to a single domestication event that has given rise to all other cultivated varieties [85]. However, publications also show that there are some genetic differences depending on the region of cultivation, and these can be used in traditional breeding to improve cowpea varieties [2,87,115,116,117,118,119,120]. It is known that there are barriers to hybridization, or recombination between members of the different cultivar groups, or with wild cowpea (var. *spontanea*) in the subspecies *unguiculata* [50]. However, these mechanisms can only limit, and not prevent, gene flow within and beyond the fields. Gene flow between wild subspecies has been observed; its frequency is largely dependent on the subspecies, the size and location of the area of cultivation and the occurrence of insects [50]. In this context, it is not only direct gene flow via pollen that has to be considered, but also seed spillage, seed contamination and unintended distribution via human activities. These activities can also involve cross boundary movements: for example, seed exchange between farmers. Therefore, agricultural cultivation of the Bt cowpea will lead to the introduction of the transgenes into regional varieties, whether intentionally or unintentionally. Gene flow to wild relatives is also likely to occur in the longer term [59]. Consequently, the environmental effects of potential hybrid offspring are also relevant in this context. Relevant risks include the effects on genetic stability, gene expression, gene function, pleiotropic effects, persistence and invasiveness. Besides interactions with the environment, these biological mechanisms are known to be impacted by the genetic or epigenetic background of the genome into which the additional genes are introduced via gene flow. As suggested in a previous review [101], the biological characteristics of spontaneous hybrid offspring from transgenic plants cannot be predicted on the basis of the data from the field trials with the original events. Therefore, hybrid crossings are needed to gather more data on the impact of the various genetic backgrounds on gene expression, including crossings with *Vigna unguiculata* var. *spontanea*. Otherwise, it cannot be excluded that crossings between the GE cowpea and other varieties or wild relatives, can promote hybrid effects in ensuing generations, e.g., higher fitness and unexpected and non-predictable biological characteristics, which may disturb or disrupt agro-ecological systems. Therefore, risk assessment should consider the presence and occurrence of regional varieties and wild relatives, their biological characteristics and their genetic differences compared to the variety used for field trials and cultivation. Experimental crossings should be conducted under contained conditions to gather reliable data, e.g., in a closed greenhouse, before any field trials take place or cultivation is considered. If the cut-off criteria proposed by Bauer-Panskus et al. [101] as an additional step for the assessment of spatio-temporal controllability had been applied in this case, it is highly likely that the outcome would have shown sufficiently reliable risk assessment to be impossible. 

In this context, it has to be taken into account that Nigeria maintains the largest cowpea germplasm collection at the International Institute of Tropical Agriculture (IITA), with more than 15,000 landraces and over 2000 wild relatives [2]. Agricultural practices, such as re-sowing the harvest, exchanging seeds and also storage and transport of seeds, will allow the transgenic seed and its offspring to survive and persist in regional seed collections, landraces and potentially also in the collections of the IITA. According to the *African Network of Expertise* [93], wild cowpea has to be considered to be an invasive and allelopathic species with a lengthier seed dormancy in comparison to cultivated varieties. This underlines the risk of gene flow from Bt cowpea to wild cowpea becoming a pathway for replacing and displacing natural populations as well as regional varieties of cultivated cowpea, with potentially disruptive effects to ecosystems beyond the fields. This is especially relevant since West Africa is a center of biological diversity for cowpea. The fact that Bt cotton is spreading in Mexico [121] underlines the need for the precautionary principle to be applied for GE plants with a potential to persist and propagate in the environment, and thus become a threat to the center of biodiversity of the same plant species. A detailed investigation of this case has revealed disturbances in the interactions between the transgenic offspring of Bt cotton and their environment. The plants exhibit characteristics of invasive plants, such as changes in defense mechanisms to fight herbivores, which could not be predicted on the basis of the intended trait. These findings have serious implications for the protection of wild cotton species because Mexico is one of the centers of origin for cotton.

The applicant also does not sufficiently substantiate the claims that there is no threat of gene flow and potential spread of the Bt cowpea to biological diversity, traditional crops, farmers’ varieties and sustainable agriculture. Such statements also appear to ignore the risks to the importance of traditional seed saving for agro-biodiversity, seed bank collections, informed choice of breeders and farmers, food sovereignty and organic farming systems. The common heritage of Nigerian farmers and breeders who have cultivated cowpea for thousands of years is likewise put at risk.

### 3.6. The NBC Risk Assessment

The risk assessment performed by the National Biosafety Committee (NBC) suffers from several deficiencies; it did not call the statements made by the applicants into question and it appears that no attempt was made to request further data. There might be several explanations for these findings. For example, risk assessment of genetically engineered plants in the EU involves experts from several institutions: it is not only the EFSA, but also experts of the member states who contribute and comment on the risk assessment of genetically engineered plants. This approach allows, for example, the identification of data gaps or uncertainties which could otherwise escape attention. Furthermore, transgenic plants, before being applied for EU market approval, will very often have already undergone prior assessment by authorities in other states, e.g., in the US. However, in the case of the transgenic cowpea, it was the first time that an application for commercial cultivation had to be assessed. Finally, the risk assessment standards in the EU are more detailed compared to those in the Cartagena Biosafety Protocol. In this regard, the CPB might need further refinements which also might be beneficial for future updates of the Nigeria biosafety guidelines [7]. 

Clearly, it has to be acknowledged that market approval in Nigeria was only given for a limited period of time and that it will expire at the end of 2022 [3]. Thus, there is room for further considerations and improvements before further decisions are taken. Other countries, such as Ghana, where cultivation of Bt cowpea is under discussion, could certainly benefit from lessons learned in Nigeria. 

## 4. Materials and Methods

We analyzed the application for the marketing and cultivation of insect-resistant cowpea—Event AAT 709A (Ref. No. AAT/DPS-18VUIR-NG)—filed by the African Agricultural Technology Foundation and the Institute of Agricultural Research, Ahmadu Bello University Zaria [4] to the government of the Federal Republic of Nigeria in 2018, and the respective recommendation of the National Biosafety Committee (NBC) of Nigeria [3]. 

We assessed the publicly available data by taking into account the provisions of the CPB, of which both Nigeria and the EU are parties. We also referred to EU standards applied in the risk assessment of GMOs to obtain market approval [6,8] which are also in line with the Nigeria biosafety guidelines [7]. The hazards that we identified and which needed to be assessed were compared to the application [4] and the assessment made by the Nigerian authorities [29]. 

The focus of our examination was on the following topics: gene expression of the inserted genes; impact on non-target organisms; impact on bio-geochemical processes; food safety; and gene flow to other cultivated varieties or wild relatives. 

## 5. Conclusions

We conclude that the risk assessment as conducted in Nigeria is not sufficient to exclude potential adverse effects resulting from the cultivation and consumption of Bt cowpea. More data and more detailed assessment is needed to evaluate hazards in regard to the environment and biodiversity, the cowpea gene pool, the livelihoods of farmers as well as human and animal health.

In summary, the following reasons for concern were identified: (i) The expression of the r-cry1Ab gene in the GE cowpea may lack sufficient stability; (ii) The toxicity of the r-Bt proteins produced in the plants is increased by synergistic effects arising from the combination with the PI produced in the cowpea. The enhanced toxicity of the protein may affect its specificity and may be associated with unexpected adverse effects on non-target organisms; (iii) The synergies between PI and r-Bt toxins expressed in the plants may also affect microbiomes associated with cowpea, including endophytes, the mycorrhizae and other soil organisms; (iv) Synergistic effects between PI and the r-Bt toxins may also impact food safety, manifesting in enhanced immune responses, including responses to potential allergens; (v) Gene flow to regional varieties and wild relatives is likely, and there is also a substantial risk of Bt cowpea cultivation endangering biodiversity in one of the centers of origin of cowpea; (vi) The consequences of gene flow for biodiversity, seed collections and farmers may be severe. 

Based on these findings and the data available, the following worst-case scenario should be considered, individually or in combination, if Bt cowpea is cultivated long-term and on a large scale: (i) damage to biodiversity, including non-target organisms; (ii) decrease in soil fertility; (iii) increase in immune responses after consumption of cowpea; (iv) damage to the gene pool of cowpea and its wild relatives. 

These hazards and related adverse effects are plausible, and therefore the likelihood of their occurrence has to be assessed in detail. However, the risk assessment conducted in Nigeria failed to adequately address these issues. Based on the data available, we conclude that the identified risks and uncertainties are too serious and, therefore, that the risk assessment of Bt cowpea should be reviewed and amended. We further suggest suspending the authorization in Nigeria until the issues raised here have been addressed.

## Data Availability

All data required to reproduce the results presented in this study can be found in the article.
